# Acute Liver Injury Associated with Khat Use in a 24-Year-Old Male

**DOI:** 10.1155/2018/2816907

**Published:** 2018-11-21

**Authors:** Mara Waters, Adam Oxner, Sigmund Krajden, Richard Sultanian

**Affiliations:** ^1^Department of Medicine, University of Toronto, Toronto, ON, Canada; ^2^Division of Infectious Diseases, St. Joseph's Health Centre, Toronto, Ontario, Canada; ^3^Department of Laboratory Medicine and Pathobiology, University of Toronto, Ontario, Canada; ^4^Division of Gastroenterology, Department of Medicine, University of Alberta, Edmonton, AB, Canada

## Abstract

Chewing khat leaves (*Catha edulis*) is common cultural practice in Eastern African countries. Khat has been implicated in cases of acute liver injury, sometimes leading to liver failure and requiring transplantation. We report the case of a 24-year-old gentleman presenting with symptoms of acute liver failure. Bloodwork demonstrated hepatocyte-predominant liver injury. Microbiological and serological hepatitis panels were negative, and his liver biopsy demonstrated acute cholestatic hepatitis. He admitted to regular khat use for several years prior to his presentation. His liver function tests improved with cessation of khat use. This is the first reported case of acute khat-associated hepatitis in Canada. Considering cultural practices such as khat chewing in presentations of acute liver injury are important when caring for diverse patient populations.

## 1. Case Presentation

A 24-year-old male initially presented to the emergency department with a 1-week history of nausea, malaise, and jaundice. He described dark urine, pale stools, and vague abdominal pain. He had no relevant past medical history, and he denied the use of any prescription medications, nonprescription medications, or supplements. He denied any smoking, alcohol, or drug use or any exposure to environmental toxins. His family is originally from Somalia, but he was born in Canada and lived in India for 8 years of his childhood. He had no recent travel outside of Canada. On physical examination, his blood pressure was 89/49 mmHg, pulse rate was 63 beats/min, temperature was 36.7°C, respiratory rate was 16 breath/min, and oxygen saturation was 98% while breathing room air. He was alert and oriented to person, place, and time. Scleral icterus was present, and he had mild right upper quadrant tenderness with negative Murphy Sign. There was no cutaneous evidence of intravenous drug use, and there were no stigmata of chronic liver disease. The remainder of the examination was unremarkable.

Initial bloodwork demonstrated significantly elevated aminotransferase and bilirubin levels and elevated international normalized ratio (INR) (Table 1). Complete blood count, renal function and electrolytes, and additional coagulation studies were within reference ranges. Serum and urine drug screens including acetaminophen were negative. Microbiological serology was negative for hepatitis A, B, C, and E, as well as CMV, VZV, Q fever, and toxoplasmosis. Copper and iron studies were unremarkable. Immunological serology was negative for anti-mitochondrial antibody, anti-liver/kidney antibody, and anti-centromere B. Titres of anti-nuclear antibody and anti-JO-1 were weakly positive.

**Figure 1:**
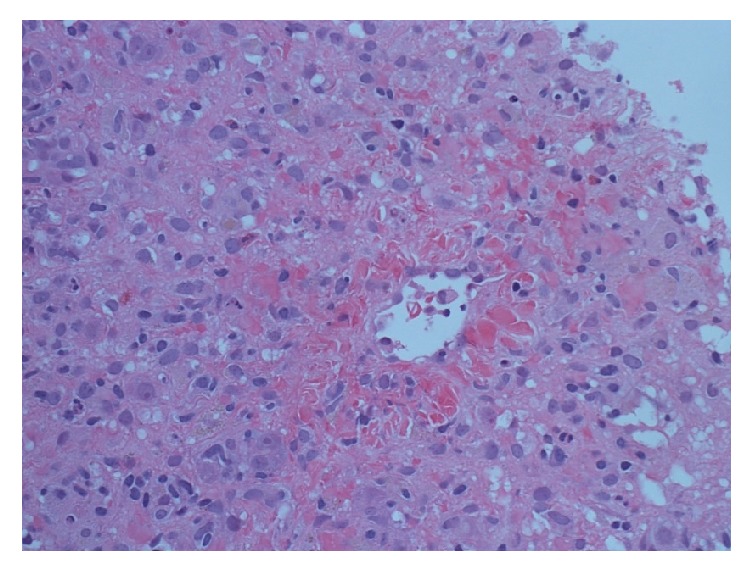
Haematoxylin and eosin stain at 400x magnification demonstrating numerous apoptotic bodies and patchy fibrotic changes in the perivenular and portal areas. This is consistent with generalized cholestatic and inflammatory changes.

**Figure 2:**
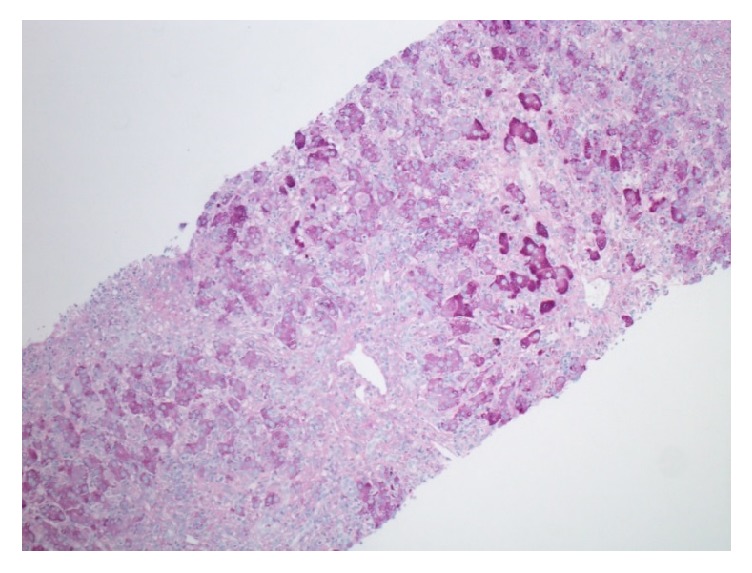
Periodic acid-Schiff stain at 100x magnification demonstrating decreased parenchymal glycogenation. PAS diastase reveals numerous Kupffer cells and portal macrophages.

An ultrasound scan of the liver was consistent with acute hepatitis and Doppler studies revealed patent hepatic and portal veins. A core needle biopsy of the liver was performed, which revealed inflammatory activity and apoptotic bodies spanning all zones of the liver, in keeping with acute cholestatic hepatitis, a pathological picture often related to drug-induced liver injury or nonhepatic viral infection (Figures 1 and 2). After investigations were complete and there was clinical and biochemical improvement, the patient was discharged from hospital on hospital day 9. However, no underlying etiology was identified.

Two months later, the patient presented to our hospital with symptoms and bloodwork similar to his original presentation (Table 1). Again, the history elicited did not reveal any new medications or exposures. However, with more directed questions regarding nonprescription medications and recreational substances, he disclosed that he engaged in weekly chewing of khat leaves for the past three years. He stated this was a cultural practice common within the Somali community, and it was easily obtainable in Canada. Given the unrevealing comprehensive diagnostic workup, it was considered that his liver injury may be related to his khat use. During the course of his admission and abstinence from khat, his transaminases improved and jaundice resolved (Table 1). He was discharged on hospital day 8. A diagnosis of khat-associated liver injury was made given the temporal relationship of use with worsening liver function, biochemical and clinical improvement with cessation of use, and the lack of evidence for an alternative diagnosis.

## 2. Discussion

Khat originates from the evergreen shrub* Catha edulis*, which is indigenous to several East African countries. Chewing the fresh leaves and shoots is a common social activity among some Somali, Ethiopian, and Yemeni cultures, as it produces euphoric symptoms similar to amphetamine ingestion [1]. This practice is also common to Somalis abroad; for example, it is estimated that 34% of Somalis living in the United Kingdom chew khat [2].

The main psychoactive agent in khat is cathinone (S-(−)-*α*-aminopropiophenone), which is metabolized to the less potent chemical R,S-(−)-norephedrine. Khat leaves are consumed fresh because cathinone dimerizes days after harvesting, which inactivates its psychoactive effects [2]. Cathine (S,S-(+)-norpseudoephedrine) is a less potent component of khat [3]. These chemicals are pharmacologically similar to amphetamine. They exert sympathomimetic effects including central nervous system stimulation, namely, euphoria and increased alertness, with maximal plasma levels being reached at 2-3 hours after chewing. Cathinone is predominantly metabolized by the liver and is fully eliminated after about 5 hours [3]. The subsequent physiological effects after elimination include depression, anorexia, and insomnia. Mood disorders and psychosis are recognized complications of chronic khat use. Case reports suggest that other possible complications may include myocardial infarction, oral cancer, and liver failure [1].

To our knowledge, this is the first reported case of acute khat-associated hepatitis in Canada. The mechanism of khat-associated hepatotoxicity is not well elucidated, though animal studies demonstrate fibrosis and liver injury with chronic use [4]. The injury is typically hepatocellular causing raised transaminase enzymes and has in some cases induced an autoimmune hepatitis wherein autoantibodies develop [5]. Resolution of the hepatitis typically occurs with cessation of use; however, there are some reports of patients developing severe acute hepatitis resulting in death or requiring liver transplantation [4, 6]. Several other case reports have been published, implicating khat use in acute hepatitis cases in Somalian immigrants [5, 7, 8]. Patients typically presented after years of use, but some cases presented after chewing for only a few weeks. There has also been suggestion that liberal use of pesticides or other contaminants during farming may contribute to the hepatotoxic effect of khat [9]; however, most studies conclude that further research is required to elucidate the mechanism of hepatotoxicity [10]. There are no published reports on pesticide measurements of Canadian khat.

In Canada, products of* Catha edulis* including cathinone are classified as a Schedule IV substances by the Controlled Drugs and Substances Act, meaning it is illegal to seek out the substance, but possession is not an indictable offense. However, many Eastern African cultures do not consider khat to be drug [2] and may not consider it relevant when discussing medication or substance use history. Therefore, it may be difficult to discover its use on a patient history without a high index of suspicion. As in the case of our patient, he only disclosed his khat use after explicit questioning on his second admission to hospital three months from his initial presentation.

Other nonnative cultural substances that have been associated with acute liver injury include Kava (*Piper methysticum*) and Kratom (*Mitragyna speciosa*). Kava is a herbal extract with anxiolytic properties used ceremonially and recreationally in the South Pacific and typically causes hepatocellular pattern of liver injury. Kratom is an herbal extract with opioid activity used recreationally in Southeast Asia and its pattern of liver injury is cholestatic or mixed [10]. Our patient had no history of ingestion of these or other substances prior to presentation.

In summary, this is the first reported case of khat-associated hepatitis in Canada which we are aware of. We identified a temporal association between khat use and liver injury followed by biochemical improvement with cessation of use in our patient, while extensive investigations were unrevealing for an alternate cause. Patients with unexplained liver injury should be asked about all medications, supplement use, alternative medical treatments, and recreational drug use. Particularly, as in our case, patients of East African heritage presenting with unexplained liver injury should be asked about khat use. This case highlights the importance of awareness of non-native cultural practices when managing patients given increased refugee and migrant populations to Canada.

## Figures and Tables

**Table 1:** Liver enzymes and liver function studies during the patient's 2 hospital admissions.

	Admission 1			Admission 2		
	Day 1	Day 3	Day 8	Day 1	Day 3	Day 8
AST (reference range [RR] 10-45 U/L)	1388	1169	886	1484	1373	646
ALT (RR 15-50 U/L)	2449	2284	1531	1827	1669	914
Total bilirubin (RR 2-20 umol/L)	190	289	254	285	220	110
INR (RR 0.8-1.2)	1.6	1.6	1.6	1.9	1.6	1.6
